# Deep Brain Stimulation for Medication Refractory Tremor in Leber Optic Neuropathy Plus Syndrome

**DOI:** 10.7759/cureus.58255

**Published:** 2024-04-14

**Authors:** Gunjanpreet Kaur, Yoan Ganev, Wilson Rodriguez, Shannon Tseng, Lissette Orozco, Pratap Chand

**Affiliations:** 1 Neurology, Saint Louis University School of Medicine, Saint Louis, USA; 2 Internal Medicine, St. Joseph Mercy Ann Arbor Hospital, Ann Arbor, USA

**Keywords:** lhon (leber’s hereditary optic neuropathy), movement disorders and tremors, leber hereditary optic neuropathy, deep brain simulator, essential tremor (et)

## Abstract

Leber hereditary optic neuropathy (LHON) is a mitochondrial disorder that presents with acute to subacute onset of unilateral progressive optic neuropathy, with sequential involvement of the fellow eye months to years later. The condition may be accompanied by neurological symptoms, including tremors, dystonia, seizures, or psychosis, in which case, it is termed LHON-plus. Here, we present the case of a 53-year-old man who was initially diagnosed with essential tremor but was later found to have LHON-plus after the onset of bilateral visual loss and a genetic panel. His essential tremor was refractory to standard pharmacological therapies, including propranolol, primidone, and topiramate. As a result, he elected to undergo bilateral deep brain stimulation (DBS) of the bilateral ventral intermediate nucleus of the thalamus with a dramatic improvement in symptoms. To our knowledge, this is the first case of essential tremor presenting in the context of LHON-plus to be treated successfully with DBS. While DBS has been applied in LHON-plus presenting with dystonia with limited success, our outcome suggests that there is promise in this approach and that more research is needed to evaluate it.

## Introduction

Leber hereditary optic neuropathy (LHON), first described in 1871, is a rare mitochondrially inherited disease characterized by subacute sudden painless loss of central vision that often presents in adolescence or middle adulthood [1]. The disease is due to mitochondrial base pair mutations: G11778A (guanine to adenine at position 11778), T14484C (tyrosine to cytosine), and G3460A. These mutations primarily affect respiratory chain complex I genes, including mitochondrial genes ND1, ND4, and ND6, among others [2]. It is less frequently associated with systemic pathology, including cardiac conduction defects or neurological conditions, manifesting as myelopathy, seizures, and movement disorders like dystonia, tremors, and psychiatric illness [3]. In those cases, the condition is termed LHON-plus syndrome [3]. Visual loss and other sequelae are usually permanent, although there have been cases of spontaneous remission and reports of success with the administration of Idebenone [4]. We describe a patient with hand tremors who was initially diagnosed with essential tremor and later developed vision loss. He was found to have LHON through genetic testing, leading to the final diagnosis of LHON-plus syndrome. The tremor was refractory to medications and required bilateral ventral intermediate nucleus of the thalamus (ViM) deep brain stimulation (DBS) for excellent symptomatic control. To our knowledge, this is the first case of LHON-plus presenting with intractable tremors to be treated with DBS.

## Case presentation

A 53-year-old Caucasian male developed hand tremors at a young age, which were diagnosed as essential tremor when he was 30. He had been treated with increasing doses of propranolol (up to 240 mg), primidone (200 mg daily), and topiramate (300 mg daily), but his bilateral postural and kinetic tremor were refractory and interfered with his work as a computer programmer and activities of daily living (ADL). His Fahn-Tolosa-Marin (FTM) tremor rating scale was 4.

At the age of 47 years, he developed progressively bilateral vision loss over a five-month period. Initial ophthalmological examination revealed subtle temporal pallor of the right optic disc, worsening retinal nerve fiber layer (RNFL) thinning in optical coherence tomography (OCT), and visual fields (Goldmann) showing central/centrocaecal scotomas bilaterally. MRI of the orbits with and without contrast showed subtle areas of elevated T2 signal in the intraorbital segment of both optic nerves and in the intraorbital segment of the right optic nerve. There was also a subtly elevated T2 signal in the optic chiasm on the right. Workup for infectious, autoimmune, and demyelinating etiologies of optic neuritis came back negative but a genetic panel revealed a G11778A mutation indicative of Leber hereditary optic neuropathy (LHON). He was started on Idebenone 900 mg/day as a supplement. His OCT RNFL measurements later progressed to optic nerve atrophy.

His postural and kinetic hand tremors had a frequency of 6-7 Hz bilaterally as measured with the Liftpulse app, with the left hand being worse than the right. The tremor was not well-controlled despite the administration of propranolol, topiramate, and primidone, so he underwent DBS of the bilateral ventral intermediate nucleus of the thalamus (ViM) using Boston Scientific's directional leads and Vercise rechargeable battery (Marlborough, Massachusetts, United States). The procedure was followed by sequential programming of the ViM DBS with excellent control of the hand tremors and improved ability to write, eat, drink, and type on the computer (see Video 1, which exhibits the patient's tremor improvement). His score on The Essential Tremor Rating Scale (TETRAS) dropped from 14 to 9 after the initial electrode programming, with further improvements in subsequent re-programming sessions. His final DBS setting is mentioned in Table 1 where level 3 of the implanted electrodes underwent cathodic stimulation. After successful DBS programming, his medications were tapered and primidone was discontinued.

**Video 1:** Patient video before and after DBS implantation of bilateral ViM demonstrating improvement in his tremor deep brain stimulation (DBS); ventral intermediate nucleus (ViM) of the thalamus

**Table 1:** Patient's final bilateral ViM DBS programming settings deep brain stimulation (DBS); ventral intermediate nucleus (VIM) of the thalamus; level 3 of electrode (L3); cathodic stimulation (C+); milliampere (mA); microsecond (μs); Hertz (Hz); Ohms (Ω)

Laterality of ViM	Right	Left
Electrode level that is stimulated; Ring mode	L3 – C +	L3 – C+
Current amplitude	2.2 mA	1.0 mA
Pulse width	90 μs	90 μs
Frequency	179 Hz	179 Hz
Impedance	2582 Ω	1000 Ω

## Discussion

Leber hereditary optic neuropathy (LHON) is a mitochondrial disorder characterized by retinal ganglion cell degeneration, leading to optic nerve atrophy and acute to subacute onset of central bilateral visual loss. The majority of LHON cases occur in young adult males, typically between 15 and 35 years of age, with a mean onset age of 27 years [5]. LHON-plus is associated with a wide variety of phenotypes involving the nervous system, including peripheral neuropathy, myelopathy, motor disorders, such as dystonia [6], spasticity, cerebellar ataxia, multiple sclerosis-like features, refractory epilepsy, psychiatric disturbances, and rarely, even severe neurodegenerative diseases [3,7]. It has also been documented to involve the cardiovascular system, causing arrhythmias and conduction defects such as preexcitation syndrome and cardiomyopathy [8-10].

Three major mtDNA point mutations (G3460A in ND1, G11778A in ND4, and T14484C in ND6) are responsible for more than 90% of LHON cases while only 5-10% of patients harbor different uncommon pathogenic mutations. Our patient had a G11778A mutation, but it is important to note that none of these mutations carry an increased predisposition to LHON-plus syndrome or any of the possible neurological presentations [8,11].

The multisystem features of LHON-plus may precede or present after the cardinal symptom of visual loss [12]. In our patient, tremors presented many years before optic nerve involvement, leading to the diagnosis of essential tremor before LHON-plus was considered. Outside of the context of LHON, essential tremor is unlikely to present with vision loss, but oculomotor abnormalities, such as difficulties with smooth pursuit and dysmetria of reflexive saccades, have been documented [13]. The combination of essential tremor and vision loss is highly suggestive of LHON, as the retinal ganglion cells are highly sensitive to the respiratory chain defects in the disease [14].

The MRI findings in our patient were typical of LHON, with atrophy of the optic nerve and cecocentral defects being among the most common features seen on visual field evaluation [15,16]. While the exact prevalence of LHON-plus with essential tremor remains unknown, a case series investigating 46 patients found that 19.5% of them had a postural tremor, indicating that this is not an uncommon presentation of LHON-plus [3]. Other studies suggest that tremors in the LHON population occur more frequently than in the general population, although the prevalence remains undetermined due to the limited population of LHON-plus patients [17].

While most patients with LHON presenting with a movement disorder are referred to a neurologist for management with medications, success rates have not been reported in the literature [18]. Our patient did not respond to the common medications given for essential tremor. One proposed theory that may explain this is that the essential tremor in LHON patients may, in fact, be a dystonic tremor, as the two are difficult if not impossible to distinguish clinically [17]. Deep brain stimulation (DBS) is a common approach in patients with medication-refractory essential tremor. As per our literature review, DBS has only previously been attempted for dystonia associated with LHON [11]. For the patient presented there, DBS did not provide symptomatic relief, in contrast to the stark improvement our patient experienced. This could be explained by the fact that in the above case report, the globus pallidus interna (GPi) was stimulated to target dystonia, while in our patient, the target was the ventral intermediate nucleus of the thalamus (ViM) to ameliorate tremors. There are no other studies in the literature that investigate outcomes of LHON-related movement disorders treated with DBS. Patients presenting with essential tremor have been treated with a conservative medicine-based approach. In our patient, essential tremor was refractory to medical therapy. With DBS, we were not only able to control his symptoms affecting his daily activities, but we were also able to remove his current medical therapy.

## Conclusions

This case report underscores the importance of considering deep brain stimulation (DBS) as a potential treatment option for patients with LHON-plus syndrome who experience refractory essential tremor. Despite conventional medical approaches proving ineffective for our patient, DBS yielded remarkable improvement.

Given the unpredictable onset of neurological symptoms associated with LHON, it is advisable to assess patients presenting with symptoms akin to essential tremor for DBS. Even if DBS is not immediately pursued, initiating a discussion regarding its potential risks and benefits is prudent. However, further research is imperative to determine the appropriate indications and safety profile of DBS in this patient population. Nevertheless, our report suggests promising prospects for DBS as a therapeutic intervention for medication-refractory tremors in patients with LHON-plus syndrome.
